# Nutritional Qualities of Commercial Meal Kit Subscription Services in Australia

**DOI:** 10.3390/nu11112679

**Published:** 2019-11-05

**Authors:** Alice A. Gibson, Stephanie R. Partridge

**Affiliations:** 1 Children’s Hospital Westmead Clinical School, The University of Sydney, 2145 Sydney, Australia; 2 Charles Perkins Centre, The University of Sydney, 2006 Sydney, Australia; 3 Westmead Applied Research Centre, Faculty of Medicine and Health, The University of Sydney, 2145 Sydney, Australia; stephanie.partridge@sydney.edu.au; 4 Prevention Research Collaboration, Sydney School of Public Health, The University of Sydney, 2006 Sydney, Australia

**Keywords:** meal kits, cooking, nutritional composition, nutrition, food supply, Australia

## Abstract

People are cooking at home less often and relying more on food prepared outside of the home, which is often of less nutritional value than home-cooked meals. The foodservice industry has endeavored to address barriers with the introduction of commercial meal kit subscription services (MKSSs). We aimed to assess and compare the nutritional qualities of MKSSs available in Australia. Average nutritional qualities per serve of 12 recipes (from four weekly boxes of three meals serving two people) were analyzed from five MKKSs (Dinnerly, HelloFresh™, MarleySpoon™, Pepper Leaf, Thomas Farms Kitchen). On average, MKSSs provided adequate serves of core foods, particularly of vegetables (2.3 ± 1.6–3.1 ± 1.8 serves per serve). Energy content ranged between 2891 ± 539 and 3904 kJ ± 890 per serve. All MKKSs were high in fat (39.5 ± 9.5–59.6 ± 11.2% of energy) and sodium (723 ± 404–1426 ± 688 mg per serve). All MKSSs met suggested dietary target level of dietary fiber for women, but none for men. If MKSS providers can modify recipes to reduce added salt and fat and increase dietary fiber, they have the potential to provide both men and women with nutritious meals that more closely align with the dietary guidelines for the prevention of chronic disease, especially if meals are used as an alternative to energy-dense nutrient-poor takeaway and convenience foods.

## 1. Introduction

Diets that are nutritionally adequate and well-balanced are essential for overall health and wellbeing [1]. Dietary risk factors, including low vegetable intake, low wholegrain intake, high salt intake, low fruit intake, high saturated fat intake and excess energy intake are leading risk factors in one-in-five deaths globally [2]. In 2017, 11.3 million deaths and 255 million disability-adjusted life years were attributable to dietary risk factors [3]. In Australia and other developed countries such as the United States of America (USA) and the United Kingdom (UK), the leading risk factors for non-communicable diseases are related to diet, including cardiovascular disease, type 2 diabetes, and certain types of cancer [4].

One contributing factor to poor quality diets is that people in developed countries are spending less time cooking and preparing meals at home, and more time eating out and ordering take away foods [5]. In 2015–2016, the average Australian spent AUD$80 per week eating out or ordering take away foods [6]. This figure increased for millennials aged under 35 years who spent over AUD$100 per week on meals prepared outside the home [6]. Restaurant and take away food consumption have been associated with significant increases in daily energy, sugar, saturated fat and sodium intakes [7]. Whereas, people who regularly prepare and cook meals at home consume a wider variety of healthy foods [8] and consume less energy on the occasions when they eat out [7]. Moreover, preparing and cooking meals at home is associated with higher dietary quality [9], and data from a large prospective study of over 12,000 people in France demonstrated that the preparation of meals from scratch was associated with a decreased risk of obesity over the five-year follow-up [10]. However, many people report lack of time and motivation as barriers to preparing and cooking meals at home [11,12].

The foodservice industry has endeavored to address home cooking barriers related to time and motivation with the introduction of commercial meal kit subscription services (MKSSs). MKSSs are defined as a subscription service that delivers recipes and fresh, pre-measured ingredients to cook them to the homes of subscribers regularly. MKSSs have increased in popularity in Australia, and globally. For example, major MKSSs, HelloFresh™ and MarleySpoon™, are operating in 10 and six countries, respectively [13,14]. Recently, Technomic described MKSSs as one of the most significant shifts in consumer food sourcing, with the global ‘Meal Kit’ marketing topping $1 billion in 2015 [15]. MKSSs are often marketed through new media, including social media influencers, as nutritionally balanced, healthy, sustainable and reduced food waste. Since the introduction of MKSSs, there has been no published research comparing such products and services. It is imperative consumers, and health professionals have independent and evidence-based information related to nutritional qualities to make an informed choice on whether to subscribe or recommend MKSSs. Therefore, the overall aim of this study was to assess and compare the nutritional qualities of MKSSs available in Australia. Specifically, we aimed to quantify the average serves of core food groups, energy, and macronutrient and micronutrient content per serve from a random selection of recipes from each of the MKSSs identified, in order to assess how they align with the Australian Dietary Guidelines.

## 2. Materials and Methods

### 2.1. Identification of Meal Kit Subscription Services

MKSSs were identified via a comprehensive online search using the advanced search tool and incognito mode embedded in the Google search engine between 7 and 11 January 2019. The website region was selected to ‘Australia’ to limit the search results to companies servicing Australia. Searches were conducted using relevant search terms including ‘meal kits,’ ‘meal kit delivery service,’ ‘meal delivery service,’ ‘meal kit subscription service,’ and ‘cooking kits.’ Both researchers (A.A.G., S.R.P.) conducted searches independently. Discrepancies between eligibility of MKSSs were discussed and resolved.

### 2.2. Inclusion Criteria

To be eligible for inclusion in the study, the commercial companies had to meet the following criteria: (i) classified as a MKSS, that is; a subscription service that delivers recipes and fresh, pre-measured ingredients required to cook them to the homes of subscribers on a regular basis; and (ii) available for delivery in the two most populous cities in Australia, Sydney and Melbourne, to ensure that the findings were applicable to a large proportion of the Australian population [16]. Commercial companies were excluded (i) if the service was not a subscription (i.e., once-off delivery); or (ii) did not involve the subscriber cooking (i.e., pre-prepared fresh or frozen meals).

### 2.3. Selection of Meals

For each MKSS identified, two researchers (AAG, SRP) each subscribed for two weeks (at different times) in total and ordered three meals per week for two people. These subscriptions resulted in 12 different meals from each of the MKSS identified. For MKSSs that offered customers a choice of meals, the options were numbered as they appeared on the MKSS website and then three meals were randomly selected using an online integer generator [17]. During the random selection of meals, the researchers did not include any meals that incurred an extra cost to the standard pricing. For example, HelloFresh™ “gourmet” or “lunch to dinner” meal kit options were excluded.

### 2.4. Outcome Measures

#### 2.4.1. Characteristics of Meal Kit Subscription Services

Ordering and recipe characteristics of each of the MKSSs were extracted from the company website or the recipe cards (either online or in hard copy). Ordering characteristics included subscription customization options including number of people, number of days and meals, number of options per week; ability to cater for dietary requirements; cost (as of January 2019); cost per serve and other features or points of difference between the companies. Recipe characteristics included display of nutritional information; additional nutritional information such as allergy information; time to prepare and cook recipe and number of times instructed to add salt to meal.

#### 2.4.2. Nutritional Qualities

Nutritional composition data was obtained by weighing all raw ingredients supplied per recipe. A standardized data collection method was developed and implemented by two researchers (AAG, SRP). All supplied ingredients per meal were photographed and weighed. Weights of ingredients were recorded using digital kitchen scales to the nearest 1 g and ingredients were as prepared according to the recipe. For example, onions and carrots were weighed after peeling, and if instructed, meats were weighed after visible fat was trimmed. All meals were cooked according to recipes provided. For this study, standardized portions of ingredients were assumed when non-specific measures were used in recipes such as ‘drizzle’ or ‘pinch.’ Each researcher preparing the meals considered a ‘drizzle’ of oil as one tablespoon and a pinch of salt as 0.4 g. If the type of oil was unspecified it was entered as ‘oil, vegetable, unspecified’. If a branded product was unavailable in the dataset, a nutritionally equivalent product was chosen. Two dietitians (AAG, SRP) entered each recipe into FoodWorks software version 8 (Xyris Software (Australia) Pty Ltd., Kenmore Hills, Australia; AusFoods 2015 and AusBrands 2015 nutrition composition databases) using a standardized protocol. Average nutrition content per serve of each recipe including number of serves of food groups, macronutrients, minerals and vitamins were determined per company.

### 2.5. Data Analysis

All data were checked and cleaned before analysis. Any data entry errors identified by value range and checks were corrected using source data (product photographs and recipes). Two researchers (A.A.G., S.R.P.) together cross-checked all data entry for consistency and accuracy. Data was extracted from FoodWorks software into Microsoft Excel, which was used to calculate the mean ± SD of the nutritional outcomes. In FoodWorks software food group serves are informed by the Australian Guide to Healthy Eating. For example, a serve of a ‘dark green’ vegetable is equal to 100 kJ, as per the nutritional modeling system used to inform the Australia Dietary Guidelines [18]. A full description of the groups, subgroups and serving sizes of food groups is available on their website [19]. To put the nutritional content of the meals in the context of dietary requirements, the average nutritional composition of the meals were compared to the 30% of the daily Nutrient References Values (NRVs) for adult Australian and New Zealanders aged 19 years and over [20]. This value was chosen because Australians typically eat three meals per day and therefore, as a minimum, we reasoned that a meal should provide at least a third of a person’s daily nutritional requirements. Where available, we compared to the suggested dietary target (SDT) to reduce chronic disease risk in addition to the recommended dietary intake (RDI) or adequate intake (AI). To calculate percent of energy from each of the macronutrients we used 16.7 kJ/g for carbohydrate and protein and 37.7 kJ/g for fat.

## 3. Results

### 3.1. Selection and Characteristics of Meal Kit Subscription Services

The search yielded five MKSSs: Dinnerly, HelloFresh™, MarleySpoon™, Pepper Leaf and Thomas Farms Kitchen. Table 1 provides an overview of the ordering options, costs and recipes characteristics of each of the five MKSSs. Only one MKSS included an option for one person. All others only included options for either two or four people. Two of the MKSSs allowed a minimum of two nights, the others all required a minimum of three. All companies provided at least eight options to choose from each week, with one company (MarleySpoon™) providing up to 20 choices. For Thomas Farms Kitchen, the automatic subscription services did not allow weekly selection, although if a person was not subscribed, they could choose from up to 10 meals to be delivered each week. All companies included an option for a box to automatically be vegetarian. The cost was almost identical across four of the MKSSs, with Dinnerly being substantially (~30%) cheaper than the others. The more affordable cost of Dinnerly is attributed to the exclusion of printed recipe cards with step by step photos of the recipes and also not having individually packed ingredients for each recipe. All but one company (Pepper Leaf) provided some level of nutrition information. The remaining four all provided information on energy and macronutrients (protein, carbohydrate and fat) with HelloFresh™ and Thomas Farms Kitchen also providing information on saturated fat, sugar and sodium. Thomas Farms Kitchen also included information on the number of fruit and vegetable serves. None of the MKKSs provided information on dietary fiber content. The average time to prepare each of the 12 recipes (as indicated in the recipe) ranged from 23 min for Pepper Leaf up to 37 min for HelloFresh™. On average, all MKSSs included instructions to add (or season with) salt more than once per recipe.

### 3.2. Nutritional Qualities

#### 3.2.1. Food Groups

The average number of vegetables serves per serve ranged from 2.3 for Thomas Farms Kitchen, up to 3.1 for HelloFresh™ (Table 2). The companies varied in the proportion of types of vegetables, with the majority coming from the red/orange vegetables or other (e.g., vegetables such as onion). Dinnerly contained the highest proportion of vegetables as legumes (~15%). Serves of grains ranged from 1.8 to 2.8 serves. Dinnerly had a much higher proportion of wholegrain serves (60%) compared with Thomas Farms Kitchen (22%), Pepper Leaf (11%) MarleySpoon™ (8%) and HelloFresh™ (0%). In terms of protein foods, serves of meat or alternatives ranged from 1.3 to 1.7 and dairy from 0.1 to 0.5.

#### 3.2.2. Macronutrients

**Figure 1:**
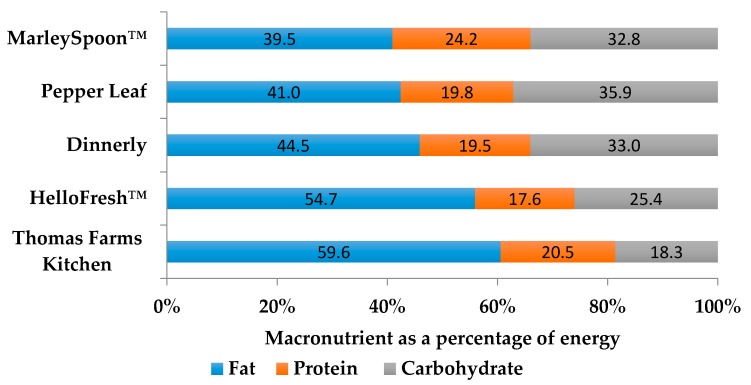
Average macronutrient profile expressed as a percentage of energy from 12 meals from each of the five meal kit subscription services.

There was substantial variability in the energy, protein, fat and carbohydrate content between, and also within, MKSSs as reflected in the relatively high standard deviations (Table 2, Figure 1). Three companies had average energy contents of ~2900 kJ per serve, whereas the other two were closer to ~4000 kJ. The higher energy content of HelloFresh™ and Thomas Farms Kitchen are due to the higher fat content, which is also reflected in a high amount of fat expressed as a percentage of energy (Figure 1). The proportion of fat as saturated fat also varied substantially from 19% for Pepper Leaf to 36% for Thomas Farms Kitchen. There was also a greater than three-fold difference in the actual quantity of saturated fat from only 6 g in MarleySpoon™ and Pepper Leaf, to 22 g in Thomas Farms Kitchen. Carbohydrate and dietary fibre content were fairly similar across MKSSs at ~55–60 g and 10 g, respectively, except for Thomas Farms Kitchen; which was lower in both carbohydrate and dietary fibre. All of the MKSSs met the AI for dietary fibre for men and women (except for Thomas Farms Kitchen for men), as well as the higher SDT level recommended level for women. However, none of the MKSS met the high SDT level of dietary fibre for men.

#### 3.2.3. Micronutrients

On average all MKSSs provided at least, or very close, to 30% of the RDI, AI or SDT for almost all included nutrients for adult males and females aged >19 years (Table 2, Supplementary Table S1). The most noteworthy exception is the high levels of sodium. All MKSSs were above 30% of the SDT for sodium for males and females. HelloFresh™ on average had almost double the sodium content (1426 mg) of the other four of the MKSSs (Table 2). None of the MKSSs met 30% of the RDI for calcium for either males or females. Pepper Leaf and Thomas Farms Kitchen were only slightly short of the RDI for magnesium, and only in men.

## 4. Discussion

This study is the first to assess and compare the nutritional qualities of MKSSs. Overall, the 60 recipes from five MKSSs analyzed in this study provided nutritious meals with adequate micronutrient content and appropriate serves of core food groups, particularly of vegetables. However, all MKSSs could benefit from changes to recipes to improve their alignment with dietary guidelines for chronic disease prevention. Specifically, by reducing or eliminating instructions for adding salt to reduce sodium; inclusion of more whole grains and legumes to increase the quantity and variety of dietary fiber; and by limiting added fat and using leaner varieties of meats or alternatives to reduce the energy, total and saturated fat content. Furthermore, all MKSSs are currently a ‘one-size-fits-all’ approach to portion size. Although commercially it may be infeasible to tailor each recipe to individual consumers energy requirements, MKKS would benefit from including information about how to modify recipes to reduce energy intake or suggest alterations in portion size for those with lower energy requirements. With these considerations and changes taken into account, MKSSs have the potential to provide both male and female consumers with nutritious meals that more closely align with dietary guidelines for prevention of chronic disease, especially if they are used as an alternative to energy-dense nutrient-poor take away and convenience foods.

Reliable and consistent evidence indicate that diets high in salt increase blood pressure, subsequently increasing the risk of cardiovascular disease [21]. A such, salt reduction has become a global public health priority, with the World Health Organization (WHO) setting salt reduction target of 30% relative reduction in mean population intakes by 2025 [22]. The WHO recommends a maximum salt intake of 5 g per day, however, the majority of countries, are exceeding this recommendation, with the estimated daily intake estimated to be 10 g/day [23]. In Australia, adult salt intake is estimated to be 9.6 g per day [24] and 64% of adults living in Australia report adding salt very often or occasionally during meal preparation or at the table [25]. One of the global salt reduction strategies is to harness industry to reduce the amount of salt in foods and meals and implement strategies to promote reformulation [22]. A study monitoring salt in ready meals in Australia found the average sodium content was 282 mg per 100 g [26]. The average serving size of a ready meal is 350 g. Therefore, the average sodium per meal was around 1000 mg sodium per serve, equating to 2.5 g of salt or half the WHO daily recommended maximum in one meal. Encouragingly, in the current study, we found all MKSSs had less salt than ready meals, with the exception of HelloFresh™, which exceeded that of ready meals with on average, 1426 mg of sodium per meal. However, only two of the MKKSs provided information on sodium. A simple strategy to reduce the sodium content of MKKS is to omit instructions to add salt in recipe preparation. MKKSs could also provide lower sodium ingredients for consumers (e.g., canned tomatoes and legumes, reduced salt soy sauces, prepared marinates, and curry bases). Sodium content should also be available for all recipes prior to ordering in order for consumers to make informed choice.

Despite the well-established health benefits of vegetable consumption, worldwide, their consumption continues to be below recommended levels. As such, increasing the consumption of vegetables is another global public health priority [27]. In Australia, the most recent data from the National Health Survey in 2017–2018 found that only 7.5% of adults aged 18 and over met the recommended 5 (or 6 for men, depending on age) serves of vegetables per day [28]. On average, men reported consuming only 2.3 serves of vegetables each day. For women it was slightly higher at 2.5 serves of vegetables per day. All MKSSs analyzed in this study, on average, provided serves of vegetables equal to or exceeding these average daily consumptions—in one meal. This suggests that in individuals who replace meals with a low vegetable content (whether home-cooked or purchased) with a MKKS meal, MKSSs may have the potential to increase the daily vegetable intake.

Furthermore, research has shown that increased confidence to prepare vegetables is related to purchasing a greater variety of vegetables, and more often [29]. MKKS may therefore also serve to increase consumers’ confidence in cooking a greater variety of vegetables and in doing so increase vegetable intake overall. However, to date, no studies have been published evaluating the use of MKKSs as an intervention to improve vegetable consumption (or overall dietary intake), but these speculations warrant investigation.

There is convincing evidence to show that higher intakes of dietary fiber are associated with a reduction in the risk of a numerous chronic diseases and their associated risk factors, as well as all-cause mortality [30]. Recent research has found that less than one in three Australian adults (28.2%) meet the AI for dietary fibre, and less than 20% of adults met the SDT to reduce the risk of chronic disease [31]. Encouragingly, we found that all MKSS on average contained at least 30% of the AI for dietary fiber (except for Thomas Farms Kitchen, which was just below for men) and all met the higher level of the SDT for females. However, none of the MKSS met the higher level of the SDT for dietary fiber for men. As well as vegetables, wholegrains and legumes are good sources of dietary fiber. Apart from Dinnerly, all other MKSSs did not contain high amounts of whole grains or legumes in the recipes randomly selected for this analysis. Of note, none of the MKKSs included dietary fiber content in their nutrition information. MKKSs would benefit from greater inclusion of whole grains and legumes to increase the variety and quantity of dietary fiber and should at least consider providing higher fiber alternatives as a customization options, e.g., brown rice instead of white rice and wholemeal pasta instead of regular pasta. Dietary fiber content should also be added to nutrition information of all MKSSs.

Over two-thirds of the population in Australia are overweight or obese [32], with similarly high rates in the USA, UK and Canada [33]. Therefore, energy intake and portion size are key considerations in dietary choices. We found that on average three of the MKSSs provided ~2900 kJ per serve and the other two provided closer to 3700–3900 kJ per serve. These figures are equivalent to approximately 33% and 45%, respectively, of an often-cited ‘average’ adult intake of 8700 kJ (~2000 kcal) [34]. However, energy requirements vary significantly between people due to differences in age, sex, body weight, body composition and activity level. Consequently, for a large proportion of the population, these energy intakes may greatly exceed an appropriate amount of energy for a person to consume in one meal, particularly if they are trying to manage their weight. All MKKSs currently follow a one-size-fits-all approach to portion size. It would be commercially infeasible for companies to individually tailor the portion (or serving) size to individual requirements. However, companies could direct their consumers to evidence-based resources (e.g., in Australia this could be on the dietary guidelines website) to help identify an appropriate portion size, as well as providing suggestions to modify recipes to reduce energy or portion size. For example, for those with lower energy requirements, they could suggest increasing the number of servings for a recipe for two to three. This increase would reduce the average energy per serve from 2900–3900 kJ to approximately 2000–2600 kJ. It may also be seen as greater value for money from the consumer perspective for a meal to provide three servings.

Further, two of the companies (Thomas Farms Kitchen and Pepper Leaf) did not provide recipes or nutritional information before selection or delivery of the meals. This information is essential for consumers to make informed dietary choices, particularly surrounding energy intake. In summary, MKKSs should ensure that energy and portion size information is available to consumers upon selection of meals and also provide consumers with information on how to modify recipes to reduce portion and energy size if needed.

Another option to reduce the energy content of the recipes is to reduce the fat content. All MKSSs were found to be high in fat (>30% of energy from fat), with two MKKSs (HelloFresh™ and Thomas Farms Kitchen) exceeding 50% of energy as fat. Reducing added fat during cooking as well as including leaner varieties of meats (where applicable) would help to reduce the total and saturated fat content. For instance, HelloFresh™ recipes did not include quantitative information for added fat and instead included frequent subjective instructions to add a ‘drizzle of olive oil’. To reduce the fat content of all MKSSs recipes, limiting the number of times fat is added during recipes or providing smaller quantitative instructions such as “teaspoons” could help to reduce the fat content, and thereby energy content. All MKKSs would benefit inclusion of leaner varieties of meats as well as instructions to trim all visible fat off meats to reduced total and especially saturated fat content. Thomas Farms Kitchen was particularly high in total and saturated fat, which is attributable to the provision of meats with very high-fat content (e.g., lamb chops and sausages). In summary, reducing the total and saturated fat content would not only reduce the energy density of the meals but would facilitate closer align with dietary guidelines.

There are limitations to this study that should be acknowledged. Firstly, our analysis is based on the selection of 12 recipes for each MKSS. However, we considered this a sufficient number to get a variety of meals, as it would represent the approximately one-month worth of meals based on three MKKS meals per week. Duplicates of each recipe were not analyzed. This potentially reduces the reliability of the results. Secondly, to minimize selection bias, we chose to randomly select meals. This method does not take into account individual preferences, which may have been a more or less nutritious option. For this reason, nutritional information for meals should be available when making choices. Thirdly, we also assumed standard quantities for adding salt and fat when instructed, and individuals may add more or less than the standard quantities we assumed. Therefore, we may have over or underestimated the average fat or salt content. Fourthly, the study was conducted over a short time period (January–March 2019), corresponding to summertime in Australia. Therefore, there may be seasonal differences in the nutritional quality of meals, which our findings do not detect. Lastly, our rationale for evaluating the nutrient content of the meals was based on meeting or exceeding 30% of the NRVs. However, meal patterns may differ substantially between and within individuals. Thus, although the average nutrient content of an average serve found in this study may fall short or exceed 30% of the NRVs, this may not reflect actual contribution to an individual’s usual intake.

The ability of MKSSs to improve the nutritional intake of consumers depends on the consumer’s prior dietary intake. If the consumers were previously consuming energy-dense nutrient-poor takeaway or convenience food, MKSSs will have a much more significant impact on dietary intake than if they were already consuming home-cooked meals using fresh ingredients. Consumers of commercial MKSSs may be limited to those of higher socioeconomic status who can afford to pay for the convenience, limiting the reach of MKSSs. Work is currently underway to understand the characteristics of consumers of MKSSs and the impact of consuming MKKSs on dietary intake and habits and cooking skills.

## 5. Conclusions

MKKSs have the potential to provide a nutritious substitute to home-cooked meals or alternative to take-away and convenience foods if several changes are made to align them more closely with dietary guidelines for the prevention of chronic disease. These changes include reducing or eliminating added salt, increasing whole grains and legumes, reducing added fat and using leaner varieties of meat. Consumers of MKSSs should be advised to avoid adding salt and limit added fat, even if they are instructed in recipes to do so. Consumers managing their weight should also consider modifying recipes to reduce portion size and energy intake.

## Figures and Tables

**Table 1:** Overview of the ordering options, costs and recipes characteristics of each of the five meal kit subscription services.

Feature	Dinnerly	HelloFresh™	MarleySpoon™	Pepper Leaf	Thomas Farms Kitchen
Customization options					
Number of people	2 or 4	2 or 4	2 or 4	1, 2, 4 or 6	2 or 4
Number of days/meals	3	3, 4 or 5	2, 3 or 4	3, 4 or 5	2, 3 or 4
Number of options per week	8	8	20	8	10 (no choice with subscription)
Dietary requirements automatically catered for ^a^	Vegetarian	Vegetarian	Vegetarian	Vegetarian	Vegetarian, OR can select no to any of: beef, lamb, pork, chicken, fish or shellfish
Cost (AUD including delivery) ^b^	47.95	69.95	69.90	69.90	69.90
Cost per serve ^c^	7.99	11.65	11.65	11.65	11.65
Other features/points of difference	No printed recipe cards or step by step photos, recipes not individually packed				
Recipes					
Were full details of recipes available when choosing weekly meal options?	Yes	Yes	Yes	No	No
Did they include nutrition information?	Per serving: energy (kJ), protein, total fat, carbohydrate	Per serving and per 100 g: energy (kJ/kcal), protein, total fat, saturated fat, carbohydrate, sugars and sodium	Per serve: energy (kcal), total fat, protein and carbohydrate	None	Per serve and per 100 g: energy (kJ), protein, total fat, saturated fat, carbohydrate, sugars, sodium and fruit/vegetable serves
Average time per recipe (minutes)	29	37	31	23	26
Average number of times instructed to add salt per recipe	1.4	2.3	1.3	1.8	1.9

^a^ This does not include dietary requirements that can be catered for by self-selecting meals based on information provided in recipes for the weekly options list. ^b^ Based on a kit for two people for three days/meals per week ^c^ Excluding any sign on discounts.

**Table 2:** Comparison of the average nutritional qualities per serve from 12 recipes from each of the five meal kit subscription services.

Nutritional Quality	Dinnerly	HelloFresh™	MarleySpoon™	Pepper Leaf	Thomas Farms Kitchen
**Food groups** (No. of serves) ^a^	Mean ± SD	Mean ± SD	Mean ± SD	Mean ± SD	Mean ± SD
Vegetable	2.6 ± 0.7	3.1 ± 1.8	2.8 ± 1.2	2.6 ± 1.3	2.3 ± 1.6
Dark green	0.3 ± 0.5	0.1 ± 0.2	0.3 ± 0.4	0.3 ± 0.4	0.1 ± 0.3
Red orange	0.6 ± 0.6	1.4 ± 1.7	0.8 ± 1.2	0.6 ± 0.5	0.8 ± 1.0
Starchy	0.1 ± 0.4	0.4 ± 0.7	0.1 ± 0.4	0.4 ± 1.1	0.2 ± 0.5
Legumes	0.4 ± 0.7	0.1 ± 0.5	0.2 ± 0.6	0.3 ± 0.6	0.1 ± 0.5
Other	1.1 ± 0.9	0.9 ± 0.7	1.4 ± 0.8	0.9 ± 0.4	1.1 ± 1.3
Grains	2.4 ± 1.9	1.8 ± 1.9	2.5 ± 1.8	2.8 ± 1.4	1.8 ± 2.0
Refined	1.5 ± 2.0	1.8 ± 1.9	2.3 ± 1.9	2.4 ± 1.6	1.4 ± 2.0
Wholegrain	0.9 ± 1.5	0.0 ± 0.0	0.2 ± 0.7	0.3 ± 1.1	0.4 ± 1.1
Meat/alternatives	1.4 ± 0.8	1.3 ± 0.7	1.7 ± 0.9	1.3 ± 0.5	1.7 ± 0.4
Dairy	0.2 ± 0.2	0.5 ± 0.6	0.2 ± 0.3	0.1 ± 0.1	0.4 ± 0.5
**Macronutrients**					
Energy (kJ)	2945 ± 873	3683 ± 787	2891 ± 539	2959 ± 930	3904 ± 890
Protein (g)	34.4 ± 14.1	38.9 ± 12.2	41.9 ± 10.5	35.0 ± 13.6	48.0 ± 8.4
Total fat (g)	34.8 ± 11.2	53.4 ± 11.8	30.3 ± 9.9	32.2 ± 15.7	61.7 ± 17.9
Saturated fat (g)	9.6 ± 6.6	16.7 ± 8.8	6.3 ± 2.1	6.0 ± 3.3	22.2 ± 12.6
Polyunsaturated (g)	5.7 ± 3.2	6.4 ± 3.0	6.0 ± 3.3	5.4 ± 3.4	5.7 ± 2.1
Monounsaturated (g)	16.9 ± 6.8	26.5 ± 7.5	15.4 ± 7.2	18.2 ± 10.1	29.1 ± 5.6
Carbohydrate (g)	58.2 ± 35.9	56.0 ± 25.8	56.8 ± 21.6	63.6 ± 20.3	42.7 ± 30.0
Sugars (g)	14.9 ± 12.0	17.0 ± 8.2	10.9 ± 4.1	10.7 ± 7.8	9.0 ± 3.7
Dietary fibre (g)	10.7 ± 3.3	10.1 ± 5.2	10.4 ± 3.0	10.2 ± 4.7	8.6 ± 3.3
**Minerals**					
Sodium (mg)	853 ± 467	1426 ± 688	779 ± 334	866 ± 488	723 ± 404
Potassium (mg)	1164 ± 222	1343 ± 337	1168 ± 222	1151 ± 563	1216 ± 337
Calcium (mg)	174 ± 91	279 ± 183	173 ± 93	179 ± 157	233 ± 146
Phosphorus(mg)	523 ± 149	605 ± 137	575 ± 130	519 ± 186	628 ± 159
Zinc (mg)	4.1 ± 1.6	4.2 ± 1.4	5.9 ± 3.1	4.1 ± 1.8	6.1 ± 2.4
Iron (mg)	4.8 ± 1.3	4.4 ± 1.4	5.1 ± 1.5	5.1 ± 1.7	5.4 ± 2.4
Magnesium (mg)	132 ± 55	124 ± 28	123 ± 45	112 ± 38	107 ± 16
**Vitamins**					
Thiamin (mg)	0.5 ± 0.3	0.6 ± 0.6	0.4 ± 0.3	0.5 ± 0.7	0.4 ± 0.3
Riboflavin (mg)	0.4 ± 0.2	0.6 ± 0.2	0.4 ± 0.1	0.4 ± 0.1	0.5 ± 0.1
Niacin (mg) ^b^	16.6 ± 7.9	18.8 ± 9.4	17.5 ± 6.7	16.0 ± 9.7	22.0 ± 5.8
Vitamin B6 (mg) ^c^	1.1 ± 0.8	0.9 ± 0.5	1.3 ± 1.3	1.0 ± 0.7	0.9 ± 0.4
Vitamin B12 (μg)	1.1 ± 1.3	1.2 ± 0.8	1.4 ± 0.8	1.3 ± 1.3	2.0 ± 0.8
Folate (μg) ^d^	201 ± 126	150 ± 122	116 ± 51	132 ± 62	95 ± 51
Vitamin C (mg)	65 ± 54	60 ± 58	80 ± 71	60 ± 38	45 ± 39
Vitamin E (mg)	7.9 ± 3.3	11.4 ± 4.6	7.6 ± 4.3	7.7 ± 3.3	9.4 ± 3.0
Vitamin A (μg) ^e^	403 ± 408	1111 ± 985	710 ± 732	487 ± 437	557 ± 561

^a^ In FoodWorks software food group serves are informed by the Australian Guide to Healthy Eating. A full description of the groups, subgroups and serving sizes of food groups is available on their website. ^b^ Niacin equivalents; ^c^ By analysis; ^d^ Total dietary folate equivalents; ^e^ Total vitamin A equivalents.

## Supplementary Materials

The following are available online at https://www.mdpi.com/2072-6643/11/11/2679/s1, Table S1: Comparison of the average nutritional content per serve from 12 meals from each of the five meal kit subscription services with nutrient reference values for Australia and New Zealanders.
