# Supplementary material for: Nutritional Qualities of Commercial Meal Kit Subscription Services in Australia
Source: Nutrients. 2019 Nov 5;11(11):2679. doi: 10.3390/nu11112679 (PMC6893801; doi:10.3390/nu11112679)
Supplement: Supplementary file 1 [file nutrients-11-02679-s001.pdf]

Table S1. Comparison of the average nutritional content per serve from 12 meals from each of the 5 Meal Kit Subscription Services with Nutrient Reference Values for Australia and New Zealanders. NRVs are for adult males and females aged >19 years. Highlighted in red are value which do not meet (or exceed) 30% of the RDI, AI or SDT.

Dietary variable	NRV classification	NRV	30% of NRV	Dinnerly	HelloFresh™	MarleySpoon™	Pepper Leaf	Thomas Farms Kitchen
				**MALES**				
Dietary fibre (g)	SDT	38	11.4	**10.7 ± 3.3**	**10.1 ± 5.2**	**10.4 ± 3.0**	**10.2 ± 4.7**	**8.6 ± 3.3**
Dietary fibre (g)	AI	30	9	10.7 ± 3.3	10.1 ± 5.2	10.4 ± 3.0	10.2 ± 4.7	**8.6 ± 3.3**
**Minerals**								
Sodium (mg)	SDT	2000	600	**853 ± 467**	**1426 ± 688**	**779 ± 334**	**866 ± 488**	**723 ± 404**
Potassium (mg)	AI	3800	1140	1164 ± 222	1343 ± 337	1168 ± 222	1151 ± 563	1216 ± 337
Calcium (mg)	RDI	1000[1]	300	**174 ± 91**	**279 ± 183**	**173 ± 93**	**179 ± 157**	**233 ± 146**
Phosphorus(mg)	RDI	1000	300	523 ± 149	605 ± 137	575 ± 130	519 ± 186	628 ± 159
Zinc (mg)	RDI	14	4.2	4.1 ± 1.6	4.2 ± 1.4	5.9 ± 3.1	4.1 ± 1.8	6.1 ± 2.4
Iron (mg)	RDI	8	2.4	4.8 ± 1.3	4.4 ± 1.4	5.1 ± 1.5	5.1 ± 1.7	5.4 ± 2.4
Magnesium (mg)	RDI	400	120	132 ± 55	124 ± 28	123 ± 45	**112 ± 38**	**107 ± 16**
**Vitamins**			0					
Thiamin (mg)	RDI	1.2	0.36	0.5 ± 0.3	0.6 ± 0.6	0.4 ± 0.3	0.5 ± 0.7	0.4 ± 0.3
Riboflavin (mg)	RDI	1.3[2]	0.39	0.4 ± 0.2	0.6 ± 0.2	0.4 ± 0.1	0.4 ± 0.1	0.5 ± 0.1
Niacin (mg)[a]	RDI	16	4.8	16.6 ± 7.9	18.8 ± 9.4	17.5 ± 6.7	16.0 ± 9.7	22.0 ± 5.8
Vitamin B6 (mg)[b]	RDI	1.3[3]	0.39	1.1 ± 0.8	0.9 ± 0.5	1.3 ± 1.3	1.0 ± 0.7	0.9 ± 0.4
Vitamin B12 (µg)	RDI	2.4	0.72	1.1 ± 1.3	1.2 ± 0.8	1.4 ± 0.8	1.3 ± 1.3	2.0 ± 0.8
Folate (µg)[c]	RDI	400	120	201 ± 126	150 ± 122	**116 ± 51**	132 ± 62	**95 ± 51**
Vitamin C (mg)	RDI	45	13.5	65 ± 54	60 ± 58	80 ± 71	60 ± 38	45 ± 39
Vitamin C (mg)	SDT	220	66	**65 ± 54**	**60 ± 58**	80 ± 71	**60 ± 38**	**45 ± 39**
Vitamin E (mg)	AI	10	3	7.9 ± 3.3	11.4 ± 4.6	7.6 ± 4.3	7.7 ± 3.3	9.4 ± 3.0
Vitamin E (mg)	SDT	19	5.7	7.9 ± 3.3	11.4 ± 4.6	7.6 ± 4.3	7.7 ± 3.3	9.4 ± 3.0
Vitamin A (µg)[d]	RDI	900	270	403 ± 408	1111 ± 985	710 ± 732	487 ± 437	557 ± 561
Vitamin A (µg)	SDT	1500	450	403 ± 408	1111 ± 985	710 ± 732	487 ± 437	557 ± 561
				**FEMALES**				
Dietary fibre (g)	SDT	28	8.4	**10.7 ± 3.3**	**10.1 ± 5.2**	**10.4 ± 3.0**	**10.2 ± 4.7**	**8.6 ± 3.3**
	AI	25	7.5	10.7 ± 3.3	10.1 ± 5.2	10.4 ± 3.0	10.2 ± 4.7	8.6 ± 3.3
**Minerals**								
Sodium (mg)	SDT	2000	600	**853 ± 467**	**1426 ± 688**	**779 ± 334**	**866 ± 488**	**723 ± 404**
Potassium (mg)	AI	3800	1140	1164 ± 222	1343 ± 337	1168 ± 222	1151 ± 563	1216 ± 337
Calcium (mg)	RDI	1000[1]	300	**174 ± 91**	**279 ± 183**	**173 ± 93**	**179 ± 157**	**233 ± 146**
Phosphorus(mg)	RDI	1000	300	523 ± 149	605 ± 137	575 ± 130	519 ± 186	628 ± 159
Zinc (mg)	RDI	8	2.4	4.1 ± 1.6	4.2 ± 1.4	5.9 ± 3.1	4.1 ± 1.8	6.1 ± 2.4
Iron (mg)	RDI	18[4]	5.4	**4.8 ± 1.3**	**4.4 ± 1.4**	**5.1 ± 1.5**	**5.1 ± 1.7**	5.4 ± 2.4

Magnesium (mg)	RDI	310[5]	93	132 ± 55	124 ± 28	123 ± 45	112 ± 38	107 ± 16
**Vitamins**			0					
Thiamin (mg)	RDI	1.1	0.33	0.5 ± 0.3	0.6 ± 0.6	0.4 ± 0.3	0.5 ± 0.7	0.4 ± 0.3
Riboflavin (mg)	RDI	1.1	0.33	0.4 ± 0.2	0.6 ± 0.2	0.4 ± 0.1	0.4 ± 0.1	0.5 ± 0.1
Niacin (mg)[b]	RDI	14	4.2	16.6 ± 7.9	18.8 ± 9.4	17.5 ± 6.7	16.0 ± 9.7	22.0 ± 5.8
Vitamin B6 (mg)[c]	RDI	1.3[3]	0.39	1.1 ± 0.8	0.9 ± 0.5	1.3 ± 1.3	1.0 ± 0.7	0.9 ± 0.4
Vitamin B12 (µg)	RDI	2.4	0.72	1.1 ± 1.3	1.2 ± 0.8	1.4 ± 0.8	1.3 ± 1.3	2.0 ± 0.8
Folate (µg)[d]	RDI	400	120	201 ± 126	150 ± 122	**116 ± 51**	132 ± 62	**95 ± 51**
Vitamin C (mg)	RDI	45	13.5	65 ± 54	60 ± 58	80 ± 71	60 ± 38	45 ± 39
Vitamin C (mg)	SDT	190	57	65 ± 54	60 ± 58	80 ± 71	60 ± 38	45 ± 39
Vitamin E (mg)	AI	7	2.1	7.9 ± 3.3	11.4 ± 4.6	7.6 ± 4.3	7.7 ± 3.3	9.4 ± 3.0
Vitamin E (mg)	SDT	14	4.2	7.9 ± 3.3	11.4 ± 4.6	7.6 ± 4.3	7.7 ± 3.3	9.4 ± 3.0
Vitamin A (µg)[e]	RDI	700	210	403 ± 408	1111 ± 985	710 ± 732	487 ± 437	557 ± 561
Vitamin A (µg)[e]	SDT	1200	360	403 ± 408	1111 ± 985	710 ± 732	487 ± 437	557 ± 561

NRV, Nutrient Reference Value; STD, suggested dietary target; AI, adequate intake. [a] Niacin equivalents [b] By analysis [c] Total dietary folate equivalents [d] total Vitamin A equivalents. [1] For men aged > 70 years and women aged >50 years, the RDI is 1300mg, none of the MKSSs would meet 30% of this higher level either. [2] For men aged >70 years, the RDI for riboflavin is 1.6mg, Dinnerly, MarleySpoon™ and PepperLeaf would not meet 30% of this higher level. [3,4] For men and women aged > 50 years, the RDI for vitamin B6 is 1.7mg and 1.5mg respectively, all MKSS would exceed 30% of these higher values.[4]For women aged >50 years the RDI for iron is only 8mg, all MKSS would meet 30% of this value. [5] For women aged 19-30 years the RDI for magnesium is 310mg, all MKSS would meet 30% of this lower value.
